# Secondary Cutaneous Plasmacytoma as a Manifestation of Extramedullary Multiple Myeloma: A Case Report

**DOI:** 10.7759/cureus.106207

**Published:** 2026-03-31

**Authors:** Martha Daniela Alcazar-Olaiz, Axel R Marquez-Nuñez, Rosalinda Peñaloza Ramirez, José Antonio Aguilar Hidalgo

**Affiliations:** 1 Faculty of Medicine, Universidad de Guanajuato, Mexico City, MEX; 2 Molecular Biology, Hospital General Dr. Manuel Gea Gonzalez, Mexico City, MEX; 3 Dermatology, National Autonomous University of Mexico, Mexico City, MEX; 4 Pathology, Hospital Regional de Alta Especialidad de Oaxaca, Mexico City, MEX; 5 Hematology, General Hospital "Dra. Matilde Petra Montoya Lafragua", Mexico City, MEX

**Keywords:** cutaneous plasmacytoma, extramedullary multiple myeloma, monoclonal gammopathy, multiple myeloma, plasma cell dyscrasias, skin involvement

## Abstract

Multiple myeloma (MM) is a clonal plasma cell malignancy that typically remains confined to the bone marrow; however, a subset of patients develops extramedullary disease (EMD), which is associated with advanced disease stage and poor prognosis. Cutaneous involvement represents a particularly rare manifestation of EMD, accounting for a small proportion of cases and usually indicating aggressive systemic progression. Due to its infrequency and heterogeneous clinical presentation, cutaneous plasmacytoma is often underrecognized, leading to diagnostic delays. We report the case of a 54-year-old Mexican woman with a history of MM who developed a painful, violaceous, ulcerated plaque on the left upper extremity following interruption of systemic therapy due to treatment-related neuropathy. Histopathological examination revealed a dense dermal infiltrate of mature plasma cells characterized by eccentric nuclei, basophilic cytoplasm, and clock-face chromatin. Immunohistochemical analysis demonstrated strong positivity for CD138 and kappa light-chain restriction, confirming the diagnosis of secondary cutaneous plasmacytoma associated with MM. This case highlights the importance of maintaining a high index of suspicion for atypical cutaneous lesions in patients with MM, particularly in the context of disease relapse or treatment interruption. Prompt skin biopsy with immunophenotypic confirmation is essential for accurate diagnosis and timely management. Cutaneous involvement reflects aggressive disease biology and underscores the limitations of localized therapies, emphasizing the need for systemic treatment strategies. Increased awareness and reporting of such cases are crucial to improving diagnostic recognition, refining prognostic assessment, and optimizing therapeutic decision-making in MM patients with extramedullary cutaneous manifestations.

## Introduction

Multiple myeloma (MM) is a plasma cell neoplasm characterized by clonal proliferation of terminally differentiated B cells within the bone marrow, monoclonal immunoglobulin production, and progressive end-organ damage. Although MM classically remains centered in the bone marrow, a subset of patients develops extramedullary disease (EMD), reflecting the ability of malignant plasma cells to grow outside the marrow microenvironment. Reported incidence varies according to the definition used and the disease setting. In a major review focused on soft-tissue plasmacytomas, extramedullary involvement has been reported in 7-18% of patients at diagnosis and in up to 20% at relapse, whereas other series using stricter definitions have reported lower frequencies, including approximately 3.4% at diagnosis and around 5% at relapse or progression [1-3].

The pathogenesis of EMD is complex and not yet fully understood. Available evidence supports a central role for loss of dependence on the bone marrow niche, with decreased adhesion molecule expression, downregulation of chemokine receptors, and additional microenvironmental and molecular changes that facilitate plasma cell migration, tissue invasion, and survival at distant sites [1,2]. Clinically, this pattern of spread is important because EMD is consistently associated with aggressive biology, treatment resistance, and inferior outcomes [2,3].

Cutaneous involvement is one of the rarest manifestations of MM and has been reported in approximately 1%-4% of patients with plasma cell myeloma [4]. It usually appears late in the disease course and is generally regarded as a marker of advanced systemic tumor burden and poor prognosis rather than isolated local relapse [4-6]. Clinically, lesions are heterogeneous and may present as papules, nodules, plaques, or tumors with erythematous to violaceous coloration, often mimicking infectious, inflammatory, or other neoplastic dermatoses [5,6]. Because of this variability, diagnosis depends on prompt clinicopathologic correlation, including histopathologic confirmation of plasma cell infiltration and immunophenotypic support of plasma cell lineage [4-6].

Recognition of cutaneous MM has significance beyond dermatologic diagnosis. In the largest contemporary clinicopathologic series, cutaneous involvement was associated with aggressive biologic features, advanced-stage disease, and short survival after lesion onset [4]. In this context, the present case is clinically noteworthy because the cutaneous lesion developed after interruption of anti-myeloma therapy due to treatment-related neuropathy and became a clinically overt manifestation of extramedullary reactivation. This sequence underscores the need for early biopsy of atypical skin lesions in patients with MM, particularly when systemic treatment has been interrupted or disease control is uncertain [4-6].

## Case presentation

**Figure 1:**
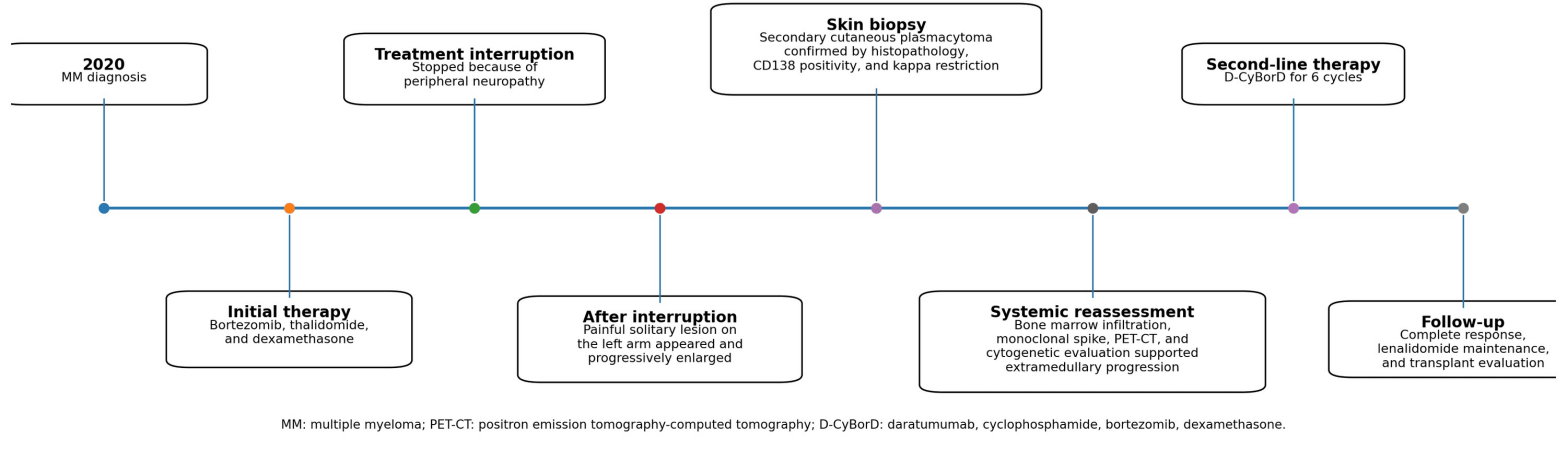
Clinical timeline of disease progression, diagnostic workup, and treatment.

**Figure 2:**
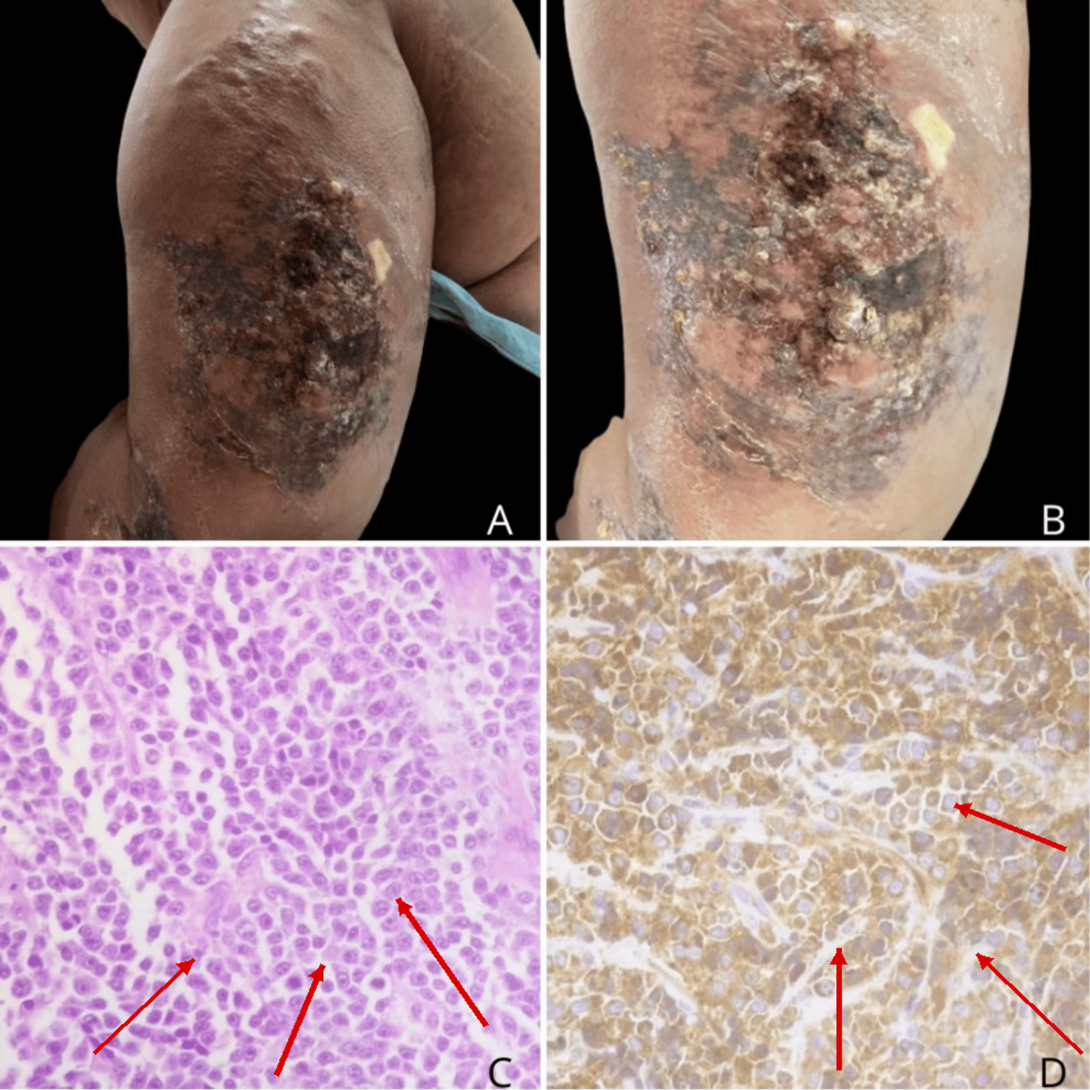
Clinical and histopathologic findings. (A, B) Solitary violaceous erythematous infiltrated plaque with ulceration, crusting, and necrotic-hemorrhagic areas on the left arm. (C) Dermal infiltrate of neoplastic plasma cells with abundant basophilic cytoplasm, eccentric nuclei, and clock-face chromatin. (D) Immunohistochemistry showing diffuse CD138 positivity and kappa light-chain restriction.

**Figure 3:**
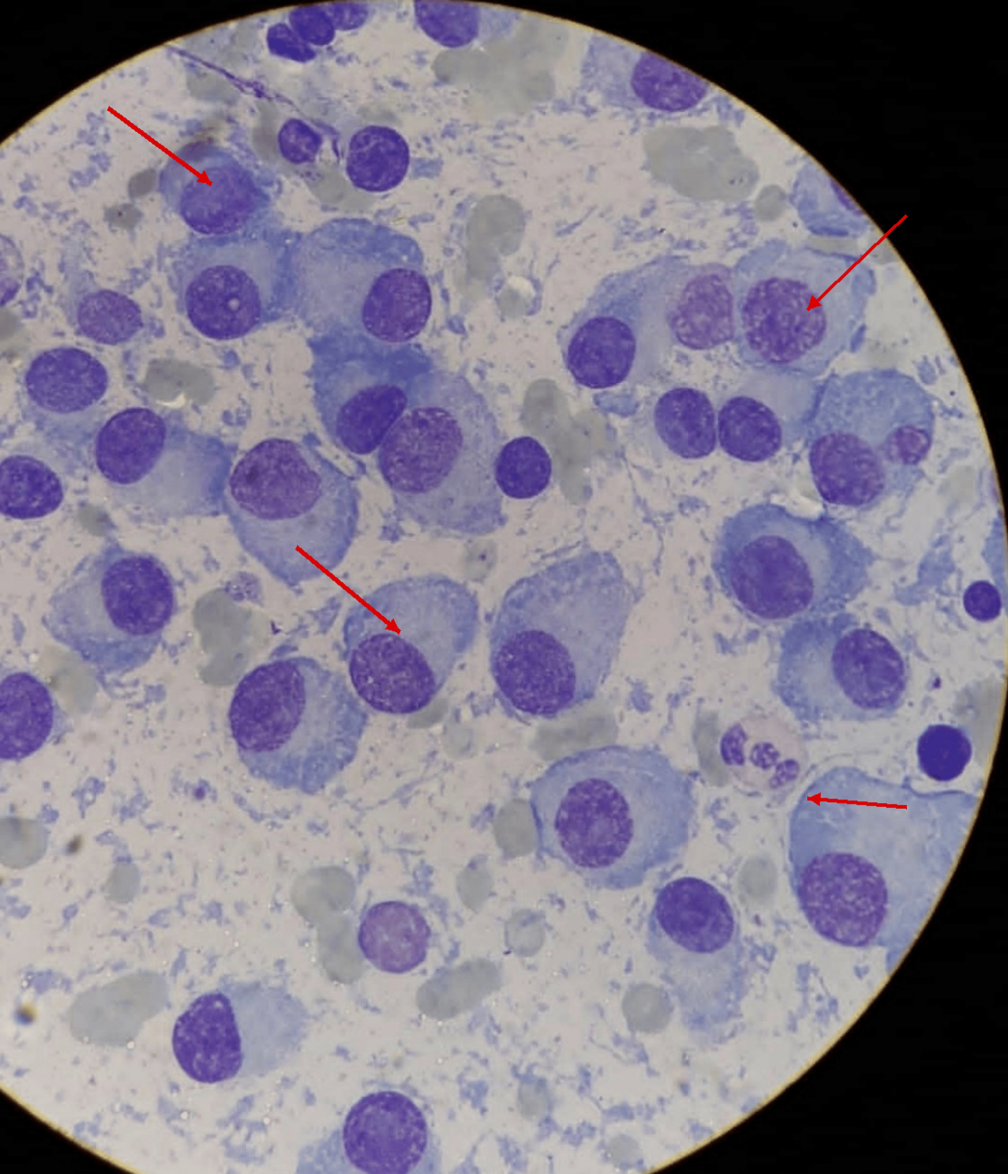
Plasma cell infiltration in bone marrow aspiration

**Figure 4:**
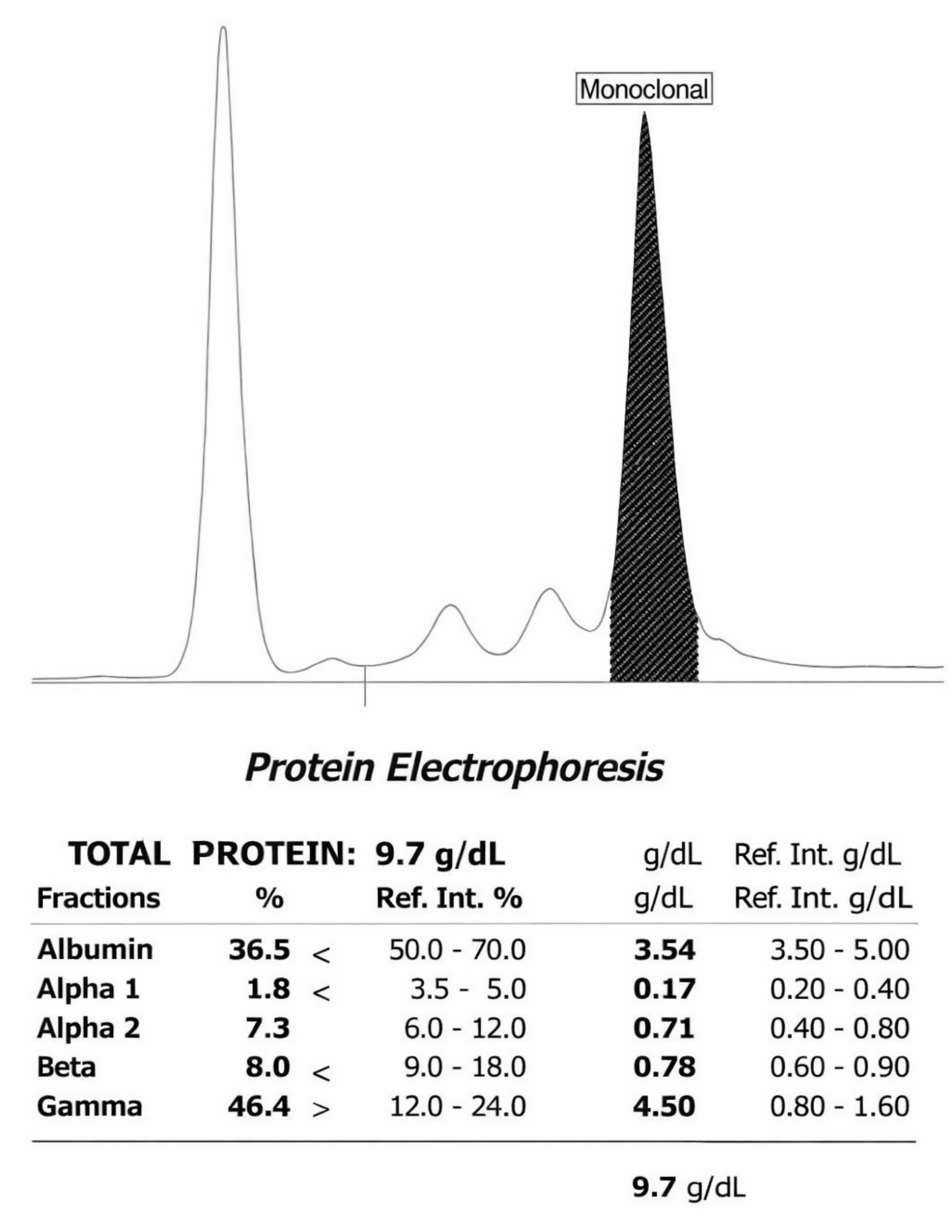
Serum protein electrophoresis demonstrating a monoclonal spike.

A 54-year-old Mexican woman with MM, diagnosed in 2020, had initially received bortezomib, thalidomide, and dexamethasone. Therapy was subsequently discontinued because of peripheral neuropathy (Figure 1). Thereafter, she developed a painful cutaneous lesion on the left arm that progressively enlarged. On physical examination, a solitary 20 × 10 cm violaceous erythematous plaque was observed on the lateral aspect of the left arm, with marked infiltration, poorly defined borders, and surrounding edema (Figure 2A, 2B). The lesion had a tumoral appearance, with extensive adherent crusting, multifocal superficial ulceration, and necrotic-hemorrhagic areas. In the setting of interrupted anti-myeloma treatment, these features raised concern for an infiltrative neoplastic process. Skin biopsy demonstrated a dense dermal infiltrate of mature plasma cells with abundant basophilic cytoplasm, eccentric nuclei, and characteristic clock-face chromatin, with inconspicuous nucleoli (Figure 2C). Immunohistochemical staining showed diffuse CD138 expression and kappa light-chain restriction (Figure 2D), confirming secondary cutaneous plasmacytoma. Subsequent systemic reassessment, including bone marrow aspiration, serum protein electrophoresis, immunofixation, serum free light-chain assay, PET-CT, and cytogenetic studies, demonstrated persistent active MM, including bone marrow plasma cell infiltration (Figure 3) and a monoclonal spike on serum protein electrophoresis (Figure 4), consistent with extramedullary progression.

Second-line treatment with D-CyBorD was initiated for six cycles, achieving a complete response. At the time of writing, the patient remains on lenalidomide maintenance therapy and is undergoing evaluation for bone marrow transplantation.

## Discussion

Cutaneous involvement in MM is an uncommon manifestation of extramedullary disease and usually reflects advanced tumor burden rather than an isolated dermatologic event [4-7]. Most reported patients develop skin lesions in the setting of active or relapsed systemic disease, and survival after cutaneous involvement is often limited, underscoring its value as a marker of biologically aggressive myeloma [4-7]. In this context, the present case is clinically relevant because the skin lesion was not an incidental finding, but rather the visible expression of systemic reactivation after interruption of anti-myeloma therapy and its temporal association. 

A major strength of this case is the clinicopathological sequence that links treatment history, lesion development, tissue diagnosis, and systemic restaging. The patient had established MM, subsequently interrupted therapy because of peripheral neuropathy, and thereafter developed a solitary painful violaceous infiltrated plaque on the left arm that progressively enlarged and acquired ulcerative and necrotic-hemorrhagic surface changes. This temporal sequence is important because it places the cutaneous lesion within a biologically plausible context of relapse, rather than as an unrelated cutaneous process. Histopathologic examination demonstrated a dense dermal infiltrate of mature plasma cells, while immunohistochemistry showed diffuse CD138 expression and kappa light-chain restriction, confirming secondary cutaneous plasmacytoma. Once the skin biopsy established plasma cell infiltration, the additional findings of persistent bone marrow plasma cell involvement and a monoclonal spike on serum protein electrophoresis supported a unified interpretation of the case as extramedullary progression of MM rather than localized skin-limited disease [1-6].

The dermatologic morphology in this patient is also informative. Reported cutaneous lesions in MM most commonly include papules, nodules, plaques, or tumoral lesions with erythematous to violaceous coloration, often with rapid growth and occasional ulceration [4-7]. Our patient showed a solitary 20 × 10 cm violaceous erythematous infiltrated plaque with poorly defined borders, edema, extensive crusting, and multifocal superficial ulceration. This phenotype falls within the recognized morphologic spectrum of cutaneous MM, but its large plaque-like and ulceronecrotic appearance broadens the practical differential diagnosis, which may include infection, pyoderma gangrenosum, and other neutrophilic ulcerative dermatoses, vasculopathic lesions, cutaneous lymphoma, or other metastatic neoplasms. For that reason, prompt biopsy remains essential in patients with MM who develop atypical or rapidly progressive skin lesions [4-6].

This case also highlights the importance of integrating dermatopathology with systemic disease assessment. In MM with suspected extramedullary spread, skin biopsy alone is not sufficient, because the clinical implication of the lesion depends on whether it represents direct extension from adjacent bone disease or true extramedullary dissemination associated with broader hematogenous escape from the marrow compartment [1-3,8]. PET/CT is particularly useful in this setting because it can identify metabolically active extramedullary sites, refine disease burden assessment, and contribute prognostic information during both restaging and response evaluation [8,9]. Thus, in the present case, the combination of cutaneous histology, bone marrow reassessment, serum monoclonal studies, and whole-body imaging provided a more cohesive interpretation of disease progression and increased the scientific value of the case beyond a purely descriptive dermatologic report.

From a therapeutic standpoint, secondary cutaneous plasmacytoma should generally be approached as a manifestation of systemic MM rather than as a purely local skin tumor [1-3,6]. Although local radiotherapy may provide palliation or local control in selected symptomatic lesions, especially when pain, bleeding, or ulceration predominate, it does not address the underlying systemic clone when cutaneous disease occurs in the context of relapsed myeloma [10]. For that reason, the decision in this patient to proceed with systemic second-line therapy was clinically appropriate.

The use of D-CyBorD in this case deserves specific comment. This regimen combines a proteasome inhibitor, an alkylating agent, dexamethasone, and anti-CD38 therapy, thereby providing systemic disease control in a setting of marrow and extramedullary involvement. Although evidence specifically focused on extramedullary MM remains limited compared with conventional marrow-confined disease, emerging prospective and phase II data support daratumumab-containing combinations in this high-risk setting [11,12]. In the EMN19 study, daratumumab plus bortezomib, cyclophosphamide, and dexamethasone showed encouraging activity in patients with MM and PET/CT-confirmed extramedullary plasmacytomas, including meaningful complete response rates and PET/CT metabolic responses [11]. Likewise, daratumumab-based salvage combinations have shown activity in relapsed/refractory MM with extramedullary disease [12]. In our patient, the achievement of complete response after six cycles suggests that aggressive systemic re-treatment can still induce meaningful control even after clinically overt cutaneous relapse.

The favorable response in this case should nevertheless be interpreted with caution. Cutaneous involvement in MM remains associated overall with aggressive disease biology, and even when an initial response is achieved, long-term control may be challenging [4,6,7]. This is why the current plan of lenalidomide maintenance and evaluation for bone marrow transplantation is clinically coherent with the high-risk implications of extramedullary involvement. Rather than representing a contradiction to the known poor prognosis of cutaneous MM, the present case emphasizes the importance of early recognition and timely systemic intervention before further disease dissemination occurs.

Overall, this case reinforces three clinically relevant points. First, atypical violaceous, infiltrated, ulcerated, or tumoral skin lesions in patients with MM warrant prompt biopsy. Second, the dermatologic finding should be interpreted together with marrow, serologic, and imaging data to distinguish isolated local disease from systemic extramedullary progression. Third, when cutaneous plasmacytoma emerges after treatment interruption, it may serve as the first clinically overt sign of relapse and should prompt rapid hematologic reassessment and escalation of systemic therapy.

## Conclusions

Cutaneous plasmacytoma is a rare manifestation of MM that may represent clinically overt extramedullary progression. In this patient, the appearance of a solitary painful violaceous ulcerated plaque after treatment interruption served as the first visible sign of disease reactivation. Because such lesions may mimic inflammatory, infectious, or other neoplastic disorders, prompt biopsy with histopathologic and immunohistochemical correlation is essential for accurate diagnosis. This case underscores the need to carefully investigate new atypical skin lesions in patients with MM, particularly when systemic therapy has been interrupted or disease control is uncertain, as early recognition may facilitate timely restaging and therapeutic decision-making.
